# JCM’s Clinical Veterinary Microbiology section moves to *ASM Animal Microbiology*

**DOI:** 10.1128/jcm.00484-26

**Published:** 2026-04-30

**Authors:** Romney M. Humphries

**Affiliations:** 1 Vanderbilt University Medical Center 12328, Nashville, Tennessee, USA

## Abstract

The *Journal of Clinical Microbiology* will transition its Clinical Veterinary Microbiology section to *ASM Animal Microbiology* to strengthen focus on animal-associated microbes and One Health. This editorial highlights key contributions from the new journal and outlines phased submission changes, emphasizing collaboration between human and veterinary microbiology to address antimicrobial resistance and emerging infectious threats.

## EDITORIAL

The year 2025 brought in an exciting new addition to the American Society for Microbiology (ASM) family of journals with the launch of the open-access *ASM Animal Microbiology* (1). This new journal is focused on animal-associated microbes and their impact on animal and public health. The topics covered by the journal will be particularly impactful to our clinical microbiology community, as we adapt our laboratories towards the inexorably shifting landscape of infectious threats faced by our patients. Insights learned from the pages of *ASM Animal Microbiology* will inevitably apply to our clinical microbiology laboratories—the question is not “if” but “when.”

This premise is loud and clear in the inaugural edition of *ASM Animal Microbiology*. Kruger-Haker et al. (2), all authors who contribute to the Clinical and Laboratory Standards Institute (CLSI) Veterinary Antimicrobial Susceptibility Testing (AST) committee—the veterinary equivalent to the well-known M100 document for human pathogens (3)—discuss the challenges of susceptibility testing of bovine and porcine *Pasteurellaceae. Pasteurella* spp. are well-known, if relatively infrequent, visitors to the bacteriology bench in most human clinical microbiology laboratories. Collaboration between this group and the CLSI AST Subcommittee led to updates in the forthcoming M45 4th Edition breakpoints for *Pasteurella* spp. that cause infections in humans. Alignment on methods between veterinary and human clinical microbiology laboratories on testing of these important zoonotic pathogens will strengthen our collective response to antimicrobial threats in these pathogens, improving the care of both human and animal patients.

Ewbank et al. then bring in the contributions of wildlife to the One Health antimicrobial resistance (AMR) cycle (4). AMR carriage by wildlife has been well-documented, occurring via selective pressure imposed from antibiotic contamination of their environment and/or exposure to AMR microbes from human and livestock waste. However, whether wildlife can then serve as a reservoir, spreader of AMR, or possibly an “incubator” for novel AMR following exposure to antimicrobials in their environment is unknown. This is not an angle often considered by most clinical microbiologists, but it is clearly an opportunity for collaboration across the field of microbiology. At the very least, awareness by the clinical microbiologist of these AMR issues, as they emerge, will better prepare us for when they inevitably appear in our own laboratories. But even more importantly, awareness of these issues will enable us to participate in the mitigation of AMR in our environments, via collaborative antimicrobial and diagnostic stewardship, curtailed use of antimicrobials in veterinary medicine and animal production systems, and advocacy for a One Health approach to AMR.

Also in the inaugural issue of *ASM Animal Microbiology*, Savin et al. explore, through core-genome analysis, the relationship between *Yersinia enterocolitica* infections in humans and isolates from pigs and cattle (5). The insights from this work point to unique transmission routes between porcine and bovine strains to humans, which persist over decades, identifying clear public health targets for prevention of disease in the human population.

Veterinary microbiology topics, exemplified by those above, have traditionally been covered in a section of the *Journal of Clinical Microbiology* (JCM), reflecting the significant technical and conceptual overlap between human and veterinary clinical microbiology. One of the goals for the creation of *ASM Animal Microbiology* was to provide a strong voice for the veterinary microbiology community (1). As such, with the launch of the new journal, the Clinical Veterinary Microbiology section of JCM will be transferred to *ASM Animal Microbiology*. This transition highlights the editorial coordination across ASM Journals and will enable a more focused use of one of our most precious resources—our reviewers—while ensuring the rigor of animal microbial science through editorial expertise.

The transfer of JCM’s clinical veterinary microbiology section to *ASM Animal Microbiology* will occur in two phases. Effective 1 April 2026, authors may select to submit veterinary papers to either the JCM or *ASM Animal Microbiology*, and the content will be considered by the journal selected by the authors. As of 1 July 2026, submissions to JCM on clinical veterinary microbiology will be redirected to *ASM Animal Microbiology*. To ensure continuity and the ongoing strength of the veterinary microbiology content in *ASM* Journals, Dr. Barbara Byrne, Editor for veterinary microbiology content in JCM, will join the Editors of *ASM Animal Microbiology* and will serve both journals during the transition. Dr. Byrne has made substantial contributions to JCM throughout her tenure as an Editor with us, and I thank her for her impeccable work on behalf of our journal. On a personal note, I will greatly miss our routine interactions, as I have learned much from her, but I look forward to her contributions to *ASM Animal Microbiology*, highlighting the veterinary microbiologist’s voice at the journal. Additionally, we have had the benefit of several editorial board members with expertise in veterinary microbiology who have made contributions through peer review of papers in this section. Thank you for your work with JCM. These members will remain on JCM’*s* Editorial board for the duration of their terms.

I am excited for this new stage with ASM Journals, and more importantly, the strong emphasis on a One Health approach to clinical microbiology as a whole between our two journals.
